# Cloud-Point Extraction Combined with Liquid Chromatography for the Determination of Ergosterol, a Natural Product with Diuretic Activity, in Rat Plasma, Urine, and Faeces

**DOI:** 10.1155/2013/479056

**Published:** 2013-04-10

**Authors:** Dan-Qian Chen, Jun-Min An, Ya-Long Feng, Ting Tian, Xiang-Yang Qin, Ying-Yong Zhao

**Affiliations:** ^1^Key Laboratory of Resource Biology and Biotechnology in Western China, Ministry of Education, The College of Life Sciences, Northwest University, No. 229 Taibai North Road, Xi'an, Shaanxi 710069, China; ^2^Department of Nephrology, Xi'an No. 4 Hospital, No. 21 Jiefang Road, Xi'an, Shaanxi 710004, China; ^3^Department of Chemistry, School of Pharmacy, Fourth Military Medical University, Xi'an, Shaanxi 710032, China

## Abstract

Ergosterol from many medicinal fungi has been demonstrated to possess a variety of pharmacological activities *in vivo* and *in vitro*. A new method based on cloud-point extraction has been developed, optimized and validated for the determination of ergosterol in rat plasma, urine and faeces by liquid chromatography. The non-ionic surfactant Triton X-114 was chosen as the extract solvent. The chromatographic separation was performed on an Inertsil ODS-3 analytical column with a mobile phase consisting of methanol and water (98 : 2, v/v) at a flow rate of 1 mL/min. The methodology was validated completely. The results indicated good performance in terms of specificity, linearity, detection and quantification limits, precision and accuracy. The method was successfully applied to the pharmacokinetic studies of ergosterol in rats. The results indicate that the ergosterol levels in feces are much higher than those in plasma and urine of the rat.

## 1. Introduction

Ergosterol is one of the best-known steroids, which exists widely in many medicinal fungi such as *Polyporus umbellatus*, *Cordyceps sinensis*, and *Hypsizigus marmoreus* [1–3]. We have recently reported the ergosterol has diuretic activity from *Polyporus umbellatus* [4]. Ergosterol has also been reported to possess cytotoxic activity [5] and anti-inflammatory activity [3]. Despite the fact that ergosterol showed multiple pharmacological activities, several pharmacokinetic and biochemical aspects of this compound remain unclear. However, the final effect of the drug *in vivo* might be influenced by many factors, such as body-and/or cell-compartment distribution, drug metabolism, lipophilicity, membrane permeability, and protein binding. So, these multiple pharmacological activities of ergosterol make it worth carrying out further study on pharmacokinetic properties and elimination pathway of ergosterol.

A number of methods have been reported for the quantification of ergosterol in raw materials, such as high performance liquid chromatography-ultraviolet detection (HPLC-UV) [1, 6–11], high performance liquid chromatography-tandem mass spectrometry (HPLC-MS/MS) [12, 13], and gas chromatography-mass spectrometry (GC-MS) [14–16]. To the best of our knowledge, there is no information describing the quantification of ergosterol in biological samples such as rat or human plasma, urine, and faeces. In addition, the effects on the elimination pathway of ergosterol have not been reported. In general, preclinical research including metabolism and pharmacokinetics of herbal medicine components are of great importance in understanding their biological effects and safety [17, 18]. In order to investigate the pharmacokinetic properties and elimination pathway of ergosterol, a simple and reproducible analytical method for quantification of ergosterol in rat plasma, urine, and faeces is required. Recently, the cloud-point extraction (CPE) method has aroused much attention as a convenient alternative to the conventional extraction systems. Compared with classical organic solvents, it offers the advantages of safety, low cost, high-concentration efficiency, easy disposal of surfactants, low toxicity, less environmental pollution, and simple procedure. CPE has been increasingly applied for the selective extraction of various analytes from biological and environmental samples (proteins, vitamins, and metal ions) [19], but only a few reports involve the extraction of drugs from plasma for pharmacokinetic studies [20, 21]. In this paper, a simple, specific, and reproducible HPLC-UV method with a simple protein precipitation procedure was described to determine ergosterol in rat plasma, urine, and faeces for the first time. The method was validated and successfully applied to the pharmacokinetic study of ergosterol in rat plasma, urine, and faeces after oral administration of ergosterol at a dose of 100 mg/kg.

## 2. Experimental

### 2.1. Chemicals and Reagents

**Figure 1:**
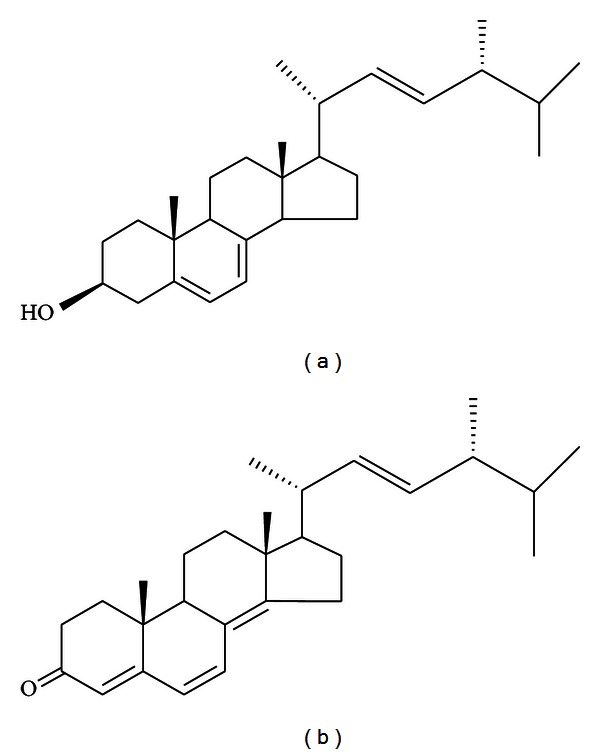
Chemical structures of ergosterol (a) and ergone (b).

The standard of ergosterol (Figure 1(a)) was isolated by the authors from *Polyporus umbellatus*. The procedure isolation and purification of ergosterol was published in our previous paper [22]. Its structure was characterized by chemical and spectroscopic methods (^1^H NMR, ^13^C NMR, and MS) and compared with those found in the literature [23]. Ergosta-4,6,8(14),22-tetraen-3-one (ergone, Figure 1(b)) was used as internal standard (IS) which was one of the components of *Polyporus umbellatus* purified in our laboratory with 99% purity as determined by HPLC. HPLC-grade methanol was purchased from Baker Company (Baker Inc., USA). Ultrahigh purity water was prepared by a Millipore-Q SAS 67120 MOLSHEIM (France).

### 2.2. Preparation of Standards and Quality Control Samples

Standard stock solutions of ergosterol (5 *µ*g/mL) and ergone (1, 5, and 100 *µ*g/mL) were prepared by dissolving suitable amounts of pure substance in acetone and were stored in darkness at 4°C.

Faecal samples were homogenized with acetone in the ratio 1 : 10 (g : vol = faeces : acetone) to obtain faecal homogenate. For HPLC determination, calibration standards of ergosterol at concentration levels of 0.09, 0.25, 0.50, 0.75, 1.00, 2.00, and 2.50 *µ*g/mL were prepared by spiking appropriate amount of the standard solutions in blank plasma and urine obtained from healthy rats. Calibration standards of ergosterol at concentrations of 0.08, 0.25, 5, 25, 50, 100, and 125 *µ*g/mL were also prepared by spiking appropriate amount of the standard solutions in faecal homogenate obtained from healthy rats. The quality control (QC) samples were separately prepared in a similar manner as those used for the calibration curve. Concentrations of 0.25, 0.75, and 2.00 *µ*g/mL were used for plasma and urine calibration standards, whereas concentrations of 0.25, 25, and 100 *µ*g/mL were used for faecal homogenate corresponding to the low QC, medium QC, and high QC, respectively.

### 2.3. Chromatographic Conditions

HPLC was performed with a Waters 2695 instrument composed of a quaternary pump, a column oven, and a Waters 2487 dual wavelength absorbance detector, and Empower was used for data collection. The chromatographic separation was performed on an Inertsil ODS-3 analytical column (250 mm × 4.6 mm i.d., 5 *µ*m, Japan) with the column temperature set at 30°C. An isocratic elution of methanol (A) and water (B) was 98 : 2 (v/v) in 23 min. The flow rate was 1.0 mL/min with detector wavelength set at 283 nm (maximum absorption wavelength of ergosterol), and the injection volume was 20 *µ*L.

### 2.4. Extraction Procedures for Plasma and Urine Sample

The plasma or urine was prepared by a cloud-point extraction method. In a 1.5 mL capped centrifugal tube, 50 *µ*L of plasma or urine was spiked with 15 *µ*L (5 *µ*g/mL for plasma, 1 *µ*g/mL for urine) of IS working solution and 135 *µ*L of 0.3 mol/L sodium chloride solution. The contents were mixed for 2 min, and then followed by the addition of 100 *µ*L of 5% Triton X-114 (v/v) aqueous solution. After that, the obtained contents were well mixed again for 5 min and then incubated in the thermostatic bath at 40°C for 20 min. After the phase separation was formed by centrifugation at 5000 rpm for 10 min, the surfactant-rich phase was obtained in the bottom of the tube by the removal of the water phase. 100 *µ*L of mobile phase was spiked to the surfactant-rich phase. Then, the contents were mixed and centrifuged at 16,000 rpm for 5 min, respectively. Most of the surfactants and coextractants such as hydrophobic proteins were precipitated in the bottom of the tube, and 20 *µ*L of supernatant fluid was injected into the HPLC system for analysis.

### 2.5. Extraction Procedures for Faeces Sample

A 100 *µ*L volume of faecal homogenate standard or sample was transferred to a 1.5 mL centrifuge tube, and then 30 *µ*L of IS working solution was spiked and vortex-mixed for 1 min. 70 *µ*L of acetone was added to the solution and vortex again for 2 min. The mixture was centrifuged at 10,000 ×g for 10 min. A 20 *µ*L of supernatant of the solution was injected into the HPLC for analysis.

### 2.6. Linearity, Limit of Detection, and Lower Limit of Quantification

 HPLC-UV method was applied for the quantification of ergosterol from rat plasma, urine, and faeces. Calibration standards of seven concentrations of ergosterol for plasma and urine (0.09, 0.25, 0.50, 0.75, 1.00, 2.00, and 2.50 *µ*g/mL) and calibration standards for faecal homogenate (0.08, 0.25, 5, 25, 50, 100, and 125 *µ*g/mL) were extracted and assayed. The linearity of the calibration curve was confirmed by plotting the peak-area ratios of ergosterol to IS versus the ergosterol concentrations with a 1/*x*-weighted least-squares linear regression analysis. The limit of detection (LOD) was considered as the final concentration producing a signal-to-noise ratio of 3. The lower limit of quantification (LLOQ) was considered as the lowest concentration on the calibration curve where precision was within 20% and accuracy was within 100 ± 20% [24].

### 2.7. Precision and Accuracy

The accuracy and intra- and interday precisions of the method were determined for ergosterol according to FDA guidance for bioanalytical method validation [24]. Accuracy and precision were assessed by the determination of QC samples at three concentration levels in three different validation assays. Accuracy was calculated by the percentage difference between the concentration of drug measured from calibration curve and that of drug added to the blank plasma, urine, and faecal homogenate. Precision was expressed by the relative standard deviation (RSD).

### 2.8. Selectivity

Selectivity of the method was assessed by analyzing five independent sources of blank plasma, urine, and faecal homogenate or plasma, urine, and faecal homogenate samples spiked with ergosterol and the IS, and observing the extent to which interferent from plasma, urine, and faecal homogenate may interfere with the analyte or IS. 

### 2.9. Recovery

The extraction recovery for plasma, urine, and faecal homogenate at three different concentrations of ergosterol was determined. The analyte/IS peak-area ratios were compared to those obtained from the direct injection of the compounds dissolved in the solutions at the same theoretical concentrations.

### 2.10. Stability

The stability of ergosterol and IS stock solutions was evaluated. Short-term stability was assessed by analyzing QC plasma, urine, and faecal homogenate samples kept at room temperature for 6 h that exceeded the routine preparation time of samples. Long-term stability was determined by assaying QC plasma, urine, and faecal homogenate samples after storage at −20°C for 30 days. Freeze-thaw stability was investigated after three freeze-(−20°C) thaw (room temperature) cycles.

### 2.11. Application to Pharmacokinetics of Ergosterol

To validate the method with real samples, a trial was undertaken to determine ergosterol in the plasma and excretion samples of healthy rats, which were administered a single dose of ergosterol orally. The study was conducted in accordance with the Regulations of Experimental Animal Administration issued by the State Committee of Science and Technology of China. All procedures and the care of the rats were in accordance with institutional guidelines for animal use in research. Rats were housed in individual metabolic cages on standard laboratory food bedding in a temperature controlled room (22 ± 2°C) with a 12 h light/dark cycle. 

Male Sprague-Dawley (SD) rats, 200 ± 10 g, fasted overnight with free access to water for at least 12 h, were dosed orally by gavages with 100 mg/kg body weight of ergosterol dissolved in plant oil as a vehicle. Rats were divided into three groups (*n* = 6) based on the time of blood sampling with two animals each. The control groups received the vehicle only. The blood samples (approximately 300 *µ*L) were collected from vena orbitalis in heparinized tubes at control and 1, 2, 3, 4, 5, 6, 8, 10, 12, 14, 16, 24, and 36 h after the administration. Samples were immediately centrifuged at 5,000 ×g for 10 min and the plasma was frozen at −20°C and stored until analysis. The estimation of ergosterol in all the samples was undertaken within 36 h of blood collection by the method described previously. 

Excretion was studied in another four groups (*n* = 6) based on the time of urine and faeces sampling with three animals each. The control groups received the vehicle only. Rats were housed with free access to food and water in individual metabolic cages, except for the final 12 h before a single oral administration of 100 mg/kg of ergosterol (access to water was ad libitum during the experiment). Faeces and urine were collected after administration in different periods (0–2, 2–4, 4–6, 6–8, 8–10, 10–12, 12–14, 14–16, 16–18, 18–24, and 24–36 h). The amount of faeces and urine collected over each period was recorded, respectively, and then urine and faeces were stored at −20°C until analysis.

Pharmacokinetics analysis was carried out by noncompartmental method with the aid of the software DAS 2.0 (issued by the State Food and Drug Administration of China for pharmacokinetic study) and pharmaceutical parameters were obtained.

The percentage of ergosterol eliminated in faeces was calculated with the accumulated ergosterol eliminated in all periods divided by administration amount (ergosterol eliminated over each period was calculated using the amount of faeces multiplied by the concentration of ergosterol in it).

## 3. Results and Discussion

### 3.1. ^1^H NMR and ^13^C NMR Data of Ergosterol

The characteristics of ergosterol are ^1^H NMR (CDCl_3_, 500 MHz) 3.63 (1H, m, H-3), 5.39 (1H, m, H-6), 5.58 (1H, m, H-7), 0.63 (3H, s, H-18), 0.95 (3H, s, H-19), 1.04 (3H, d, *J* = 6.6 Hz, H-21), 5.18 (1H, dd, *J* = 15.2, 8.0 Hz, H-22), 5.22 (1H, dd, *J* = 15.2, 8.0 Hz, H-23), 0.83 (3H, d, *J* = 6.7 Hz, H-26), 0.84 (3H, d, *J* = 6.4 Hz, H-27), 0.92 (3H, d, *J* = 6.8 Hz, H-28); ^13^C NMR (CDCl_3_, 125 MHz) 38.3 (C-1), 32.1 (C-2), 70.6 (C-3), 40.8 (C-4), 139.9 (C-5), 119.7 (C-6), 116.4 (C-7), 141.5 (C-8), 46.4 (C-9), 37.1 (C-10), 21.3 (C-11), 39.0 (C-12), 42.8 (C-13), 54.7 (C-14), 23.1 (C-15), 28.4 (C-16), 55.8 (C-17), 12.2 (C-18), 17.7 (C-19), 40.5 (C-20), 21.2 (C-21), 135.5 (C-22), 132.1 (C-23), 42.9 (C-24), 33.2 (C-25), 19.8 (C-26), 20.1 (C-27), 16.4 (C-28). The data of mass spectrum was published in our previous paper [25, 26].

### 3.2. Optimization of Chromatographic Conditions and Sample Preparation

Chromatographic optimisation of HPLC conditions was carried out with respect to mobile phase conditions (methanol-water or acetonitrile-water), stationary phase (Inertsil ODS-3 column, 250 mm × 4.6 mm i.d., 5 *µ*m or Diamonsil C18 column, 250 mm × 4.6 mm i.d., 5 *µ*m), temperature (30 or 35°C), and peak shape. The test results showed that the peak shapes of ergosterol were improved by the solvent system of methanol and water.

**Figure 2:**
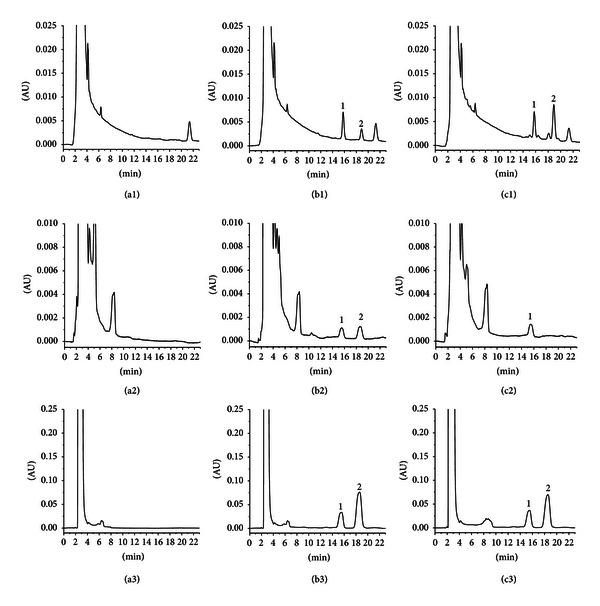
Chromatograms of ergosterol and IS in rat plasma, urine, and faecal homogenate—(a1) blank rat plasma, (a2) blank urine, (a3) blank faecal homogenate, (b1) blank plasma spiked with ergosterol and IS, (b2) blank urine spiked with ergosterol and IS, (b3) blank faecal homogenate spiked with ergosterol and IS, (c1) a rat plasma sample after oral administration, (c2) a urine sample after oral administration, and (c3) a faecal homogenate sample after oral administration (1, IS; 2, ergosterol).

The previously-mentioned method was demonstrated by comparing chromatograms of six independent biological samples (plasma, urine, and faecal homogenate) from blank rats, each as a blank sample and a spiked sample. The chromatograms of ergosterol and the IS are showed in Figures 2(a1)–2(a3), 2(b1)–2(b3), and 2(c1)–2(c3), respectively. Figures 2(b1)–2(b3) indicate no significant interferences at the retention times of ergosterol and the IS. The ergosterol and the IS of the retention times were 18.8 and 15.7 min, respectively, with a total run time of less than 23 min. System suitability parameters for the method were as follows: the theoretical plates for ergosterol and the IS were 4900 and 5400, respectively. Tailing factor was less than 1.1 for all ergosterol and the IS and resolution among ergosterol and the IS was more than 1.5. Ergosterol was chosen as IS because of its similarity to the analytes with respect to polarity, high recovery, and suitable retention time. 

### 3.3. Linearity

Calibration curves of ergosterol were linear over the concentration range of 0.09–2.50 *µ*g/mL for plasma and urine and 0.08–125 *µ*g/mL for faecal homogenate. The typical equation was *Y* = 0.8251  *X* + 0.0357 (*r* = 0.9996) for plasma, *Y* = 2.9189  *X* + 0.1265 (*r* = 0.9995) for urine, and *Y* = 0.0262  *X* + 0.0104 (*r* = 0.9994) for faecal homogenate. *Y* = peak-area ratio (ergosterol/IS) and *X* = ergosterol concentration. During routine analysis, each analytical run included a set of calibration samples, a set of QC samples, and the unknowns. The LOD of ergosterol in rat plasma, urine, and faecal homogenate were 42, 50, and 49 ng/mL, respectively. The LOQ of ergosterol in rat plasma, urine, and faecal homogenate was 90, 90, and 80 ng/mL respectively.

### 3.4. Accuracy and Precision

Table 1 shows the intra- and interday precision and accuracy for ergosterol from biological matrices QC samples. The intra- and interday precisions were measured below 9.6% and 7.8%, respectively, with relative recovery from 95.1% to 103.6%. The results indicated that the method showed good precision and accuracy.

### 3.5. Selectivity

The selectivity of the method was demonstrated by comparing chromatograms of five independent biological samples (plasma, urine, and faecal homogenate) from blank rats, each as a blank sample and a spiked sample.  Figure 2 indicates no significant interferences at the retention times of ergosterol and IS in plasma, urine, and faeces samples. The ergosterol and IS of the retention times were 18.8 and 15.7 min, respectively. 

### 3.6. Extraction Recovery

The mean extraction recovery of ergosterol from plasma, urine, and faecal homogenate was 90.9 ± 2.9%, 92.4 ± 2.6%, and 93.7 ± 3.3%, respectively. The mean relative recovery for IS was 94.5 ± 3.2% (*n* = 5). Recovery data are shown in Table 2. The results indicated that the method showed high recovery.

### 3.7. Stability

Table 3 shows the results of short-term, long-term, and freeze-thaw stability of ergosterol in plasma, urine, and faeces. Ergosterol was found to be stable in frozen plasma, urine, and faeces after three freeze-thaw cycles. The relative standard deviations were within ±15%. Long-term stability studies of ergosterol in three rat matrices showed appreciable stability over 30 days. Furthermore, ergosterol in three rat matrices was found to be stable at room temperature for a period of 6 h. All the results well met the criterion for stability measurements.

### 3.8. Application of the Method

**Figure 3:**
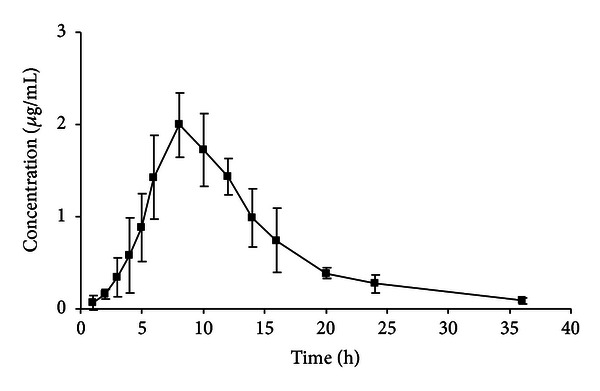
Mean (±S.D.) plasma concentration-time profile of ergosterol in the plasma of healthy rats (*n* = 6) that were administered a single oral dose of 100 mg/kg of ergosterol.

The diuretic activity of ergosterol was reported in our previous publication [4]. Pharmacokinetic studies of this drug are important for further biological and biochemical research and for future clinical trials. After a single oral administration of ergosterol (100 mg/kg) to rats, the concentrations of ergosterol in plasma, urine, and faecal homogenate were determined by the HPLC-UV method.  Figure 3 shows mean plasma concentration-time curves of ergosterol after administration. Pharmacokinetic parameters are listed in Table 4. 

**Figure 4:**
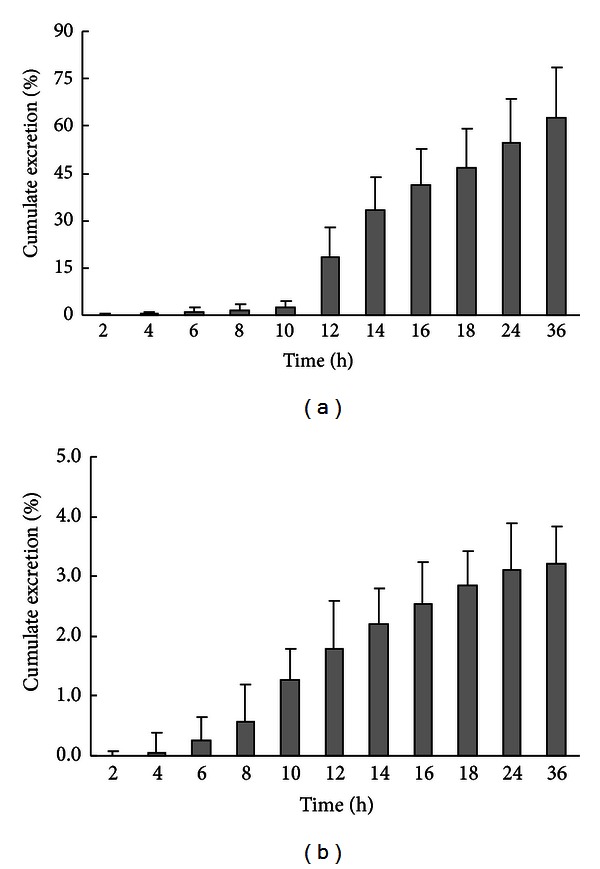
Cumulative excretion of ergosterol in faeces (a) and urine (b) of rats after an oral dose with a dosage of 100 mg/kg. Faeces were collected after administration in different periods (0–2, 2–4, 4–6, 6–8, 8–10, 10–12, 12–14, 14–16, 16–18, 18–24, and 24–36 h).

The ergosterol was detected in rat plasma, urine, and faeces samples collected from 0 to 36 h after an oral administration. The results indicate that the ergosterol levels in faeces are much higher than those in plasma and urine of the rat (Figure 4(a)). Almost 62.5% of loading dose is cumulative in the faeces within 36 h after an oral dose with a dosage of 100 mg/kg (Figure 4(a)), but the lower level of ergosterol was found in urine (Figure 4(b)).

## 4. Conclusions

The cloud-point extraction technique has been successfully applied for the first time as an effective method for the extraction and preconcentration of ergosterol from rat plasma samples. It was shown that this method is suitable for the analysis of ergosterol in rat plasma samples collected for pharmacokinetic study. The HPLC-UV method is simple, specific, and reproducible for quantitative determination of ergosterol in rat plasma, urine, and faeces. The small amount of biological matrices required (0.3 mL per determination) made this method suitable for routine analysis in preclinical pharmacokinetic studies, and the method was helpful in clinical pharmacokinetic studies. It can also be used as a reference for therapeutic drug monitoring of ergosterol.

## Figures and Tables

**Table 1:** Summary of precision and accuracy of ergosterol in rat plasma, urine, and faeces.

Added concentration (*μ*g/mL)	Found concentration (mean ± SD, *μ*g/mL)	Recovery (%)	Intraday (RSD %)	Interday (RSD %)
Plasma				
0.25	0.259 ± 0.012	103.6	9.6	7.8
0.75	0.713 ± 0.030	95.1	3.7	5.4
2.00	2.001 ± 0.072	100.1	3.9	4.8
Urine				
0.25	0.254 ± 0.009	101.6	4.2	7.1
0.75	0.736 ± 0.038	98.1	5.5	5.8
2.00	1.956 ± 0.090	97.8	3.3	3.1
Faeces				
0.25	0.247 ± 0.010	98.8	4.8	3.6
25	25.1 ± 0.7	100.4	2.8	5.1
100	99.5 ± 1.9	99.5	3.1	1.6

**Table 2:** Absolute recoveries of ergosterol in rat plasma, urine, and faeces.

Added concentration (*μ*g/mL)	Found concentration (mean ± SD, *μ*g/mL)	Recovery (%)	RSD %
Plasma			
0.25	0.227 ± 0.008	90.8	5.2
0.75	0.678 ± 0.078	90.4	2.8
2.00	1.832 ± 0.102	91.6	3.4
Urine			
0.25	0.231 ± 0.006	92.4	3.5
0.75	0.703 ± 0.065	93.7	3.8
2.00	1.825 ± 0.131	91.3	2.4
Faeces			
0.25	0.233 ± 0.009	93.2	4.7
25	23.4 ± 1.3	93.6	2.2
100	94.3 ± 3.2	94.3	2.8

**Table 3:** Summary of stability studies of ergosterol in rat plasma, urine, and faeces under various storage conditions.

Biological matrix	Added concentration (*μ*g/mL)	Found concentration (*μ*g/mL)	Recovery (%)	RSD %
	Short-term stability			

	0.25	0.249 ± 0.010	99.6	4.1
Plasma	0.75	0.714 ± 0.043	95.2	6.7
	2.00	1.90 ± 0.080	95.0	1.9
	0.25	0.252 ± 0.011	100.8	4.6
Urine	0.75	0.717 ± 0.039	95.6	1.4
	2.00	1.91 ± 0.01	95.5	1.6
	0.25	0.243 ± 0.007	97.2	2.9
Faeces	25	24.8 ± 0.63	98.8	2.5
	100	99.4 ± 2.1		2.1

	Three freeze-thaw cycles			

	0.25	0.262 ± 0.021	104.8	7.8
Plasma	0.75	0.738 ± 0.027	98.4	4.2
	2.00	1.92 ± 0.08	96.0	2.1
	0.25	0.251 ± 0.011	100.4	4.6
Urine	0.75	0.759 ± 0.032	101.2	3.5
	2.00	1.93 ± 0.04	96.5	2.2
	0.25	0.251 ± 0.007	100.4	3.2
Faeces	25	24.7 ± 0.6	98.8	2.6
	100	98.9 ± 3.3	98.9	3.3

	Long-term stability			

	0.25	0.250 ± 0.013	100.0	4.6
Plasma	0.75	0.723 ± 0.045	96.4	2.9
	2.00	1.95 ± 0.12	97.5	1.8
	0.25	0.238 ± 0.009	95.2	4.0
Urine	0.75	0.759 ± 0.007	101.2	3.1
	2.00	2.06 ± 0.15	103.0	5.2
	0.25	0.249 ± 0.011	99.6	4.5
Faeces	25	24.6 ± 0.5	98.4	2.1
	100	100.1 ± 2.4	100.1	2.4

**Table 4:** Pharmacokinetic parameters obtained after administration of ergosterol in SD rats (*n* = 6).

Pharmacokinetic parameters	mean ± SD
AUC_0–36 h_ (*μ*g h mL^−1^)	22.23 ± 5.04
*C*_*Max*⁡_ (*μ*g mL^−1^)	2.18 ± 0.21
*t*_1/2_ (h)	5.90 ± 1.43
*T*_*Max*⁡_ (h)	8.00 ± 1.26
